# Four new species and additional records of *Domene* and *Lathrobium* from the Dayao Mountains, southern China

**DOI:** 10.3897/zookeys.508.9682

**Published:** 2015-06-17

**Authors:** Zhong Peng, Zhuo Sun, Li-Zhen Li, Mei-Jun Zhao

**Affiliations:** 1 Department of Biology, Shanghai Normal University, 100 Guilin Road, Shanghai, 200234 P. R. China; 2 Dayaoshan Natural Reserve, Jinxiu Hsien, Guangxi, 545700, P. R. China

**Keywords:** Coleoptera, Staphylinidae, Paederinae, *Domene*, *Lathrobium*, new species, additional records, Dayao Mountains, China

## Abstract

Material of the paederine genera *Domene* Fauvel, 1873 and *Lathrobium* Gravenhorst, 1802 from the Dayao Mountains, southern China, is examined. Eight species are identified, three of them described previously and five undescribed. Four species are described and illustrated for the first time: *Domene hei* Peng & Li, **sp. n.**, *Lathrobium jinxiuense* Peng & Li, **sp. n.**, *Lathrobium kuan* Peng & Li, **sp. n.** and *Lathrobium leii* Peng & Li, **sp. n.** One probably undescribed species of *Lathrobium* remains unnamed.

## Introduction

Sixteen species of Lathrobiina have been reported from Guangxi (Assing 2012a, b, 2013a, 2014a, b; Feldmann et al. 2014; Li et al. 2013; Li et al. 2013a, b; Lü and Li 2014; Peng et al. 2012, 2013a, 2014a). One species of *Lobrathium* Mulsant & Rey, 1878 and two micropterous species of *Lathrobium* were previously recorded from Dayao Mountains: *Lobrathium fuscoguttatum* Li, Dai & Li, 2013 (Yinshan Station), *Lathrobium shengtangshanense* Peng & Li, 2012 (Shengtang Shan) and *Lathrobium dayaoshanense* Peng & Li, 2012 (Dayaoshan Nature Reserve), but no species of *Domene* have been reported from this mountain range.

Covering an area of 249.07 km^2^, the Dayao Mountains are situated in central Guangxi, southern China. The highest peak is the Shengtang Shan at 1,979 m. According to Deng (1984) two vertical zones of forest vegetation can be distinguished: an evergreen broad-leaved forest zone at elevations below 1,300 m and a mixed broad-leaved and coniferous forest zone at altitudes above 1,300 m.

In 2011 and 2014, Jia-Yao Hu, Zi-Wei Yin, Xiao-Bin Song, Yi-Ming Yu, Zhu-Qi Yan and the first author made two collecting trips to Dayao Mountains, where they collected numerous *Domene* and *Lathrobium* specimens. Eight species were identified, four of which are described for the first time.

## Material and methods

The following abbreviations are used in the text, with all measurements in millimeters:

Body length (BL) from the anterior margin of the mandibles (in resting position) to the abdominal apex; length of forebody (FL) from the anterior margin of the mandibles to the posterior margin of the elytra; head length (HL) from the anterior margin of the frons to the posterior margin of the head; head width (HW): maximum width of head; length of antenna (AnL); length of pronotum (PL) along midline; maximum width of pronotum (PW); elytral length (EL) at the suture from the apex of the scutellum to the posterior margin of the elytra (at the sutural angles); length of aedeagus (AL) from the apex of the ventral process to the base of the aedeagal capsule.

The type material is deposited in the Insect Collection of Shanghai Normal University, Shanghai, China (SNUC).

## Results

### Domene (Macromene) hei

Taxon classificationAnimaliaColeopteraStaphylinidae

Peng & Li sp. n.

http://zoobank.org/0452270A-1DEF-406F-8520-97BEB541A7AA

Figs 1A–C , 2

#### Type material.

Holotype: ♂, labelled ‘China: Guangxi Prov., Jinxiu Hsien, Shengtang Shan, 23°57'37"N, 110°06'46"E, 1300 m, 21.VII.2014, Peng, Song, Yan & Yu leg.’ (SNUC). Paratypes: 2 ♀♀, same data, but ‘23°59'N, 110°06'E, 1200–1400 m, 25.VII.2011, Peng, Hu & Yin leg.’ (SNUC).

#### Description.

Measurements (in mm) and ratios: BL 10.23–10.66, FL 5.67–5.84, HL 1.54–1.57, HW 1.44–1.51, AnL 3.56–3.67, PL 1.67–1.74, PW 1.39–1.44, EL 1.41–1.48, AL 1.07, HL/HW 1.04–1.07, HW/PW 1.04–1.06, HL/PL 0.90–0.92, PL/PW 1.20–1.22, EL/PL 0.84–0.86.

**Figure 1.**
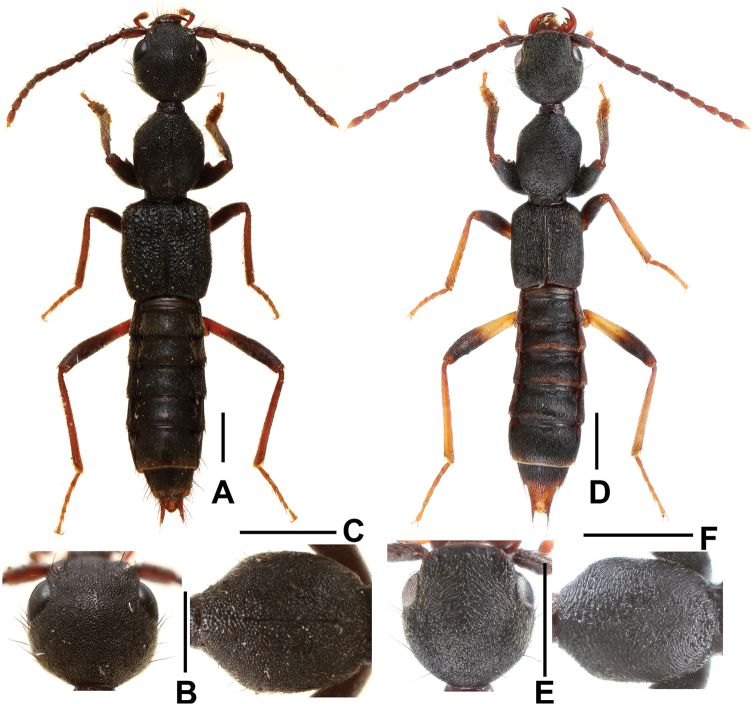
*Domene hei* (**A–C**) and *Domene chenae* (**D–F**). **A,D** habitus **B, E** head **C, F** pronotum. Scale bars: 1.0 mm.

Habitus as in Fig. 1A. Body black; legs blackish brown to brown; antennae dark brown to brown.

Head (Fig. 1B) orbicular, widest behind eyes; punctation coarse, umbilicate and dense, interstices forming narrow ridges; antenna slender.

Pronotum (Fig. 1C) somewhat narrower than head, widest in the middle; lateral margins convex in dorsal view; punctation similar to that of head; midline with rudiment of a fine glossy line.

Each elytron with more or less irregular longitudinal narrowly elevated ridges; suture elevated in posterior two thirds; macropunctation coarse and partly somewhat seriate; interstices with irregular micropunctation. Hind wings reduced. Protarsomeres I–IV distinctly dilated.

**Figure 2.**
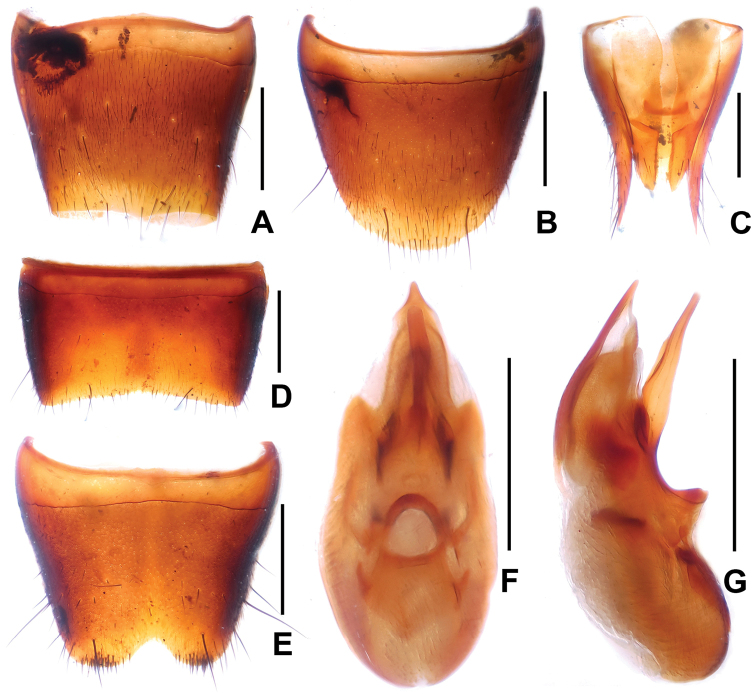
*Domene hei*. **A** female tergite VIII **B** female sternite VIII **C** female tergites IX–X. **D** male sternite VII **E** male sternite VIII **F** aedeagus in ventral view **G** aedeagus in lateral view. Scale bars: 0.5 mm.

Abdomen with fine and dense punctation on tergites III–VIII; posterior margin of tergite VIII weakly convex (Fig. 2A); interstices with distinct microreticulation; posterior margin of tergite VII with palisade fringe.

Male. Sternites III–VI unmodified; sternite VII (Fig. 2D) without modified pubescence, posterior margin broadly concave; sternite VIII (Fig. 2E) with narrow and shallow postero-median impression, posterior excision small, on either side of posterior excision with cluster of short dark setae; aedeagus as in Figs 2F, G; ventral process slender; dorsal plate with long sclerotized apical portion and short basal portion.

Female. Posterior margin of sternite VIII (Fig. 2B) broadly convex; genital segments (Fig. 2C) with a slender sclerotized structure.

#### Comparative notes.

Based on more or less irregular longitudinal elevations of elytra, the derived morphology of the aedeagus and particularly on the shapes and chaetotaxy of the male sternites VIII, *Domene hei* belongs to the *Domene scabripennis* species group. It is distinguished from other species of this group by the coloration of legs, the fine glossy line on the pronotum, the shape of the ventral process of the aedeagus and the slender sclerotized structure in the female genital segments. For illustrations of the species of the *Domene scabripennis* species group see Assing (in press a) and Assing and Feldmann (2014).

#### Etymology.

The species is dedicated to Wei-Jun He, specialist of Phasmatodea, who supported us on our field trips.

#### Distribution and natural history.

The type locality is situated in the Shengtang Shan to the southwest of Jinxiu, central Guangxi. The specimens were sifted from leaf litter in a rhododendron forest at altitudes of 1,200–1,400 m, together with *Lathrobium shengtangshanense* Peng & Li, 2012.

### Domene (Macromene) chenae

Taxon classificationAnimaliaColeopteraStaphylinidae

Peng & Li, 2014

Figs 1D–F , 3

#### Material studied.

China: Guangxi: 1 ♂, Jinxiu Hsien, Shengtang Shan, 23°57'37"N, 110°06'46"E, 1300 m, 20.VII.2014, Peng, Song, Yan & Yu leg. (SNUC); 2 ♀♀, Jinxiu Hsien, Shengtang Shan, 23°59'N, 110°06'E, 1200–1400 m, 25.VII.2011, Peng, Hu & Yin leg. (SNUC).

#### Comment.

**Figure 3.**
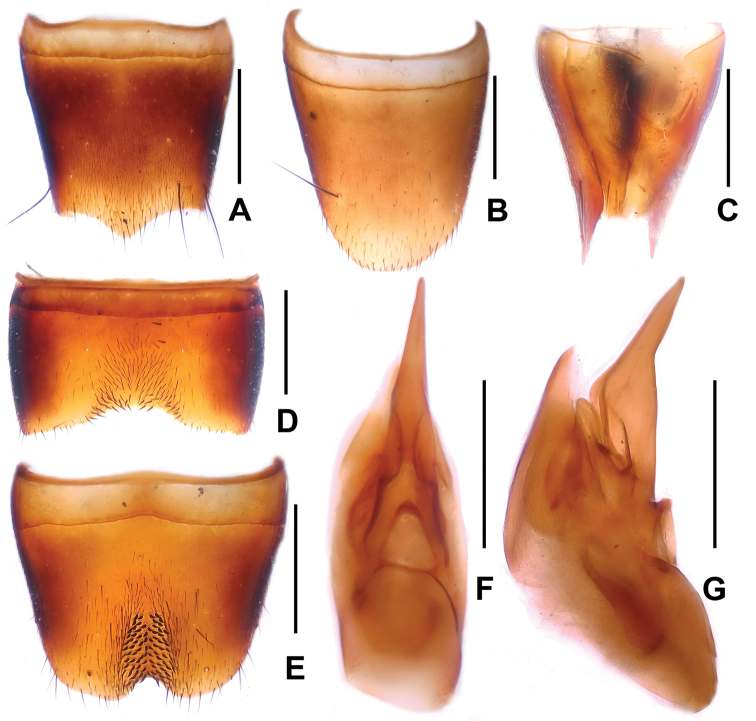
*Domene chenae* (from Shengtang Shan). **A** female tergite VIII **B** female sternite VIII **C** female tergites IX–X **D** male sternite VII **E** male sternite VIII **F** aedeagus in ventral view **G** aedeagus in lateral view. Scale bars: 0.5 mm.

*Domene chenae* was previously known only from Anjiangping in Guangxi. The above material indicates that this species is subject to considerable intraspecific variation. According to the description and illustrations provided by Feldmann et al. (2014), the distal halves of metafemora are brown (blackish brown in the material listed above, Fig. 1D) and the ventral process of aedeagus is slender (stouter in the material listed above, Figs 3F–G). For illustrations of the material from Shengtang Shan and of the type material of *Domene chenae* see Figs 1D–F, 3 and Feldmann et al. (2014), respectively.

### Lathrobium jinxiuense

Taxon classificationAnimaliaColeopteraStaphylinidae

Peng & Li sp. n.

http://zoobank.org/983EA1AD-1D31-4319-9481-CF4998D2F1BF

Figs 4A , 5

#### Type material.

Holotype: ♂, labelled ‘China: Guangxi Prov., Jinxiu Hsien, Qigongli, 24°09'07"N, 110°12'29"E, 1300 m, 16.VII.2014, Peng, Song, Yan & Yu leg.’ (SNUC). Paratypes: 1 ♀, same label data as holotype (SNUC).

#### Description.

Measurements (in mm) and ratios: BL 6.85–6.91, FL 2.96–3.06, HL 0.78–0.85, HW 0.80–0.84, AnL 1.58–1.63, PL 1.05–1.09, PW 0.84–0.87, EL 0.59–0.63, AL 0.85, HL/HW 0.98–1.01, HW/PW 0.95–0.97, HL/PL 0.74–0.78, PL/PW 1.25, EL/PL 0.56–0.58.

**Figure 4.**
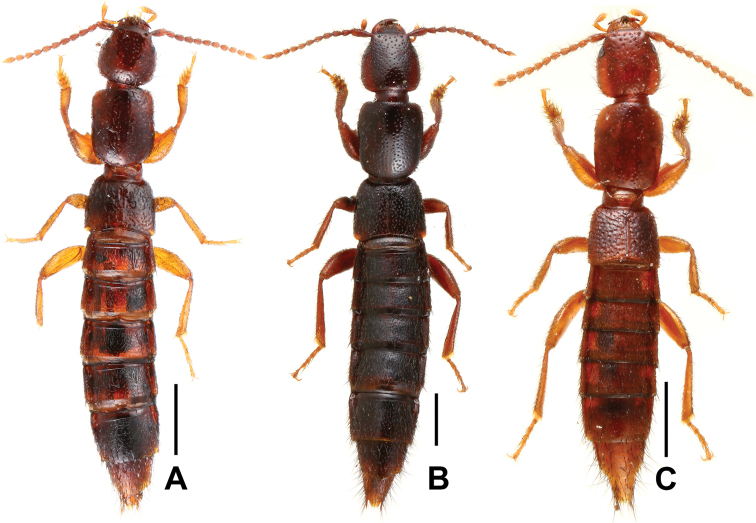
Habitus of *Lathrobium* spp., **A** *Lathrobium jinxiuense* **B** *Lathrobium kuan* **C** *Lathrobium leii*. Scale bars: 1.0 mm.

Habitus as in Fig. 4A. Body brown, legs yellowish brown, antennae light brown.

Head approximately as long as broad; punctation moderately coarse and sparse, sparser in median dorsal portion; interstices with distinct microreticulation; eyes very small and composed of approximately 20 ommatidia.

Pronotum nearly parallel-sided; punctation similar to that of head; impunctate midline broad; interstices without microsculpture.

Elytral punctation moderately dense, shallow and ill-defined. Hind wings completely reduced. Protarsi with weakly pronounced sexual dimorphism.

**Figure 5.**
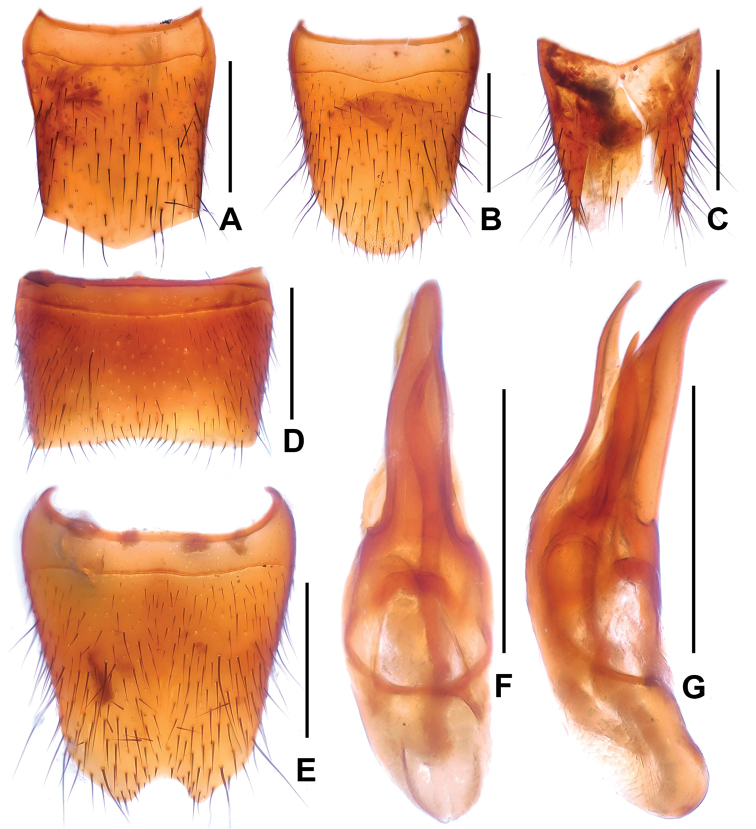
*Lathrobium jinxiuense*. **A** female tergite VIII **B** female sternite VIII **C** female tergites IX–X **D** male sternite VII **E** male sternite VIII **F** aedeagus in ventral view **G** aedeagus in lateral view. Scale bars: 0.5 mm.

Abdomen with fine and moderately dense punctation, that of tergite VII somewhat sparser than that of anterior tergites; interstices with shallow microsculpture; posterior margin of tergite VII without palisade fringe; tergite VIII without sexual dimorphism, posterior margin (Fig. 5A) obtusely angled in the middle.

Male. Sternites III-VI unmodified; sternite VII (Fig. 5D) with very shallow postero-median impression, this impression without distinctly modified setae, posterior margin weakly concave in the middle; sternite VIII (Fig. 5E) with very shallow median impression posteriorly, this impression without distinctly modified setae, posterior excision small and symmetrical; aedeagus as in Figs 5F, G, ventral process nearly symmetrical in ventral view and acute apically; dorsal plate with long and strongly sclerotized apical portion and with moderately sclerotized basal portion; internal sac with single long sclerotized spine.

Female. Posterior margin of sternite VIII (Fig. 5B) convex and with moderately dense micropubescence; tergite IX (Fig. 5C) almost completely divided; tergite X (Fig. 5C) nearly reaching anterior margin of tergite IX.

#### Comparative notes.

The new species resembles *Lathrobium maoershanense* Peng & Li, 2012 in habitus and the similarly derived morphology of the aedeagus, but differs from this species by the smaller posterior excision of the male sternite VIII, the more slender ventral process of the aedeagus and the shape of the female tergite VIII. For illustrations of *Lathrobium maoershanense* see Peng et al. (2012).

#### Etymology.

The specific epithet is derived from Jinxiu, where the type locality is situated.

#### Distribution and natural history.

The type locality is situated in Qigongli to the north of Jinxiu, central Guangxi. The specimens were sifted from leaf litter and humus in a beech forest at an altitude of 1,300 m, together with *Lathrobium dayaoshanense* Peng & Li, 2012.

### Lathrobium kuan

Taxon classificationAnimaliaColeopteraStaphylinidae

Peng & Li sp. n.

http://zoobank.org/48016D68-2999-44B6-868C-A62DCFF3F851

Figs 4B , 6

#### Type material.

Holotype: ♂, labelled ‘China: Guangxi Prov., Jinxiu Hsien, Shengtang Shan, 23°59'32"N, 110°06'26"E, 1160 m, 23.VII.2014, Peng, Song, Yan & Yu leg.’ (SNUC). Paratypes: 1 ♂, 1 ♀, same label data as holotype (SNUC).

#### Description.

Measurements (in mm) and ratios: BL 9.17–9.40, FL 4.00–4.11, HL 1.05–1.11, HW 1.05–1.15, AnL 2.00–2.15, PL 1.48–1.51, PW 1.18–1.26, EL 0.78–0.83, AL 1.90–1.93, HL/HW 0.97–1.00, HW/PW 0.89–0.91, HL/PL 0.71–0.74, PL/PW 1.20–1.25, EL/PL 0.53–0.55.

Habitus as in Fig. 4B. Body dark brown, legs and antennae brown.

Head approximately as long as broad; punctation coarse and moderately dense, somewhat sparser in median dorsal portion; interstices with shallow microreticulation; eyes small and composed of approximately 30 ommatidia.

Pronotum nearly parallel-sided; punctation similar to that of head; impunctate midline moderately broad; interstices glossy and without microsculpture.

Elytral punctation dense and well-defined. Hind wings completely reduced. Protarsi without sexual dimorphism.

**Figure 6.**
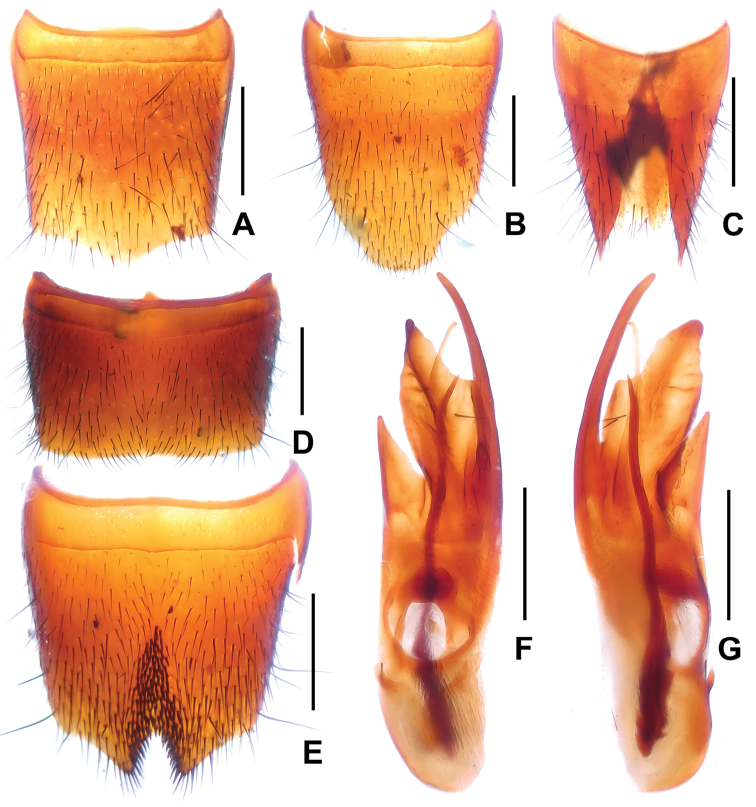
*Lathrobium kuan*. **A** female tergite VIII **B** female sternite VIII **C** female tergites IX–. **D** male sternite VII **E** male sternite VIII **F** aedeagus in ventral view **G** aedeagus in lateral view. Scale bars: 0.5 mm.

Abdomen with fine and dense punctation, that of tergite VII sparser than that of anterior tergites; interstices with distinct microsculpture; posterior margin of tergite VII without palisade fringe; tergite VIII without sexual dimorphism, posterior margin (Fig. 6A) obtusely angled in the middle.

Male. Sternites III-VI unmodified; sternite VII (Fig. 6D) with very shallow postero-median impression, this impression with weakly modified setae, posterior margin nearly truncate; sternite VIII (Fig. 6E) with very narrow median impression posteriorly, this impression with numerous short dark setae, posterior excision V-shaped and symmetrical; aedeagus as in Figs 6F, G; ventral process asymmetrical, broad and furcate; internal sac with a long strong sclerotized spine and a shorter weakly sclerotized spine.

Female. Posterior margin of sternite VIII (Fig. 6B) strongly convex; tergite IX (Fig. 6C) almost completely divided; tergite X (Fig. 6C) nearly reaching anterior margin of tergite IX.

#### Comparative notes.

Based on the modifications of the male sternite VIII, the furcate ventral process of the aedeagus, the presence of long sclerotized spines in the internal sac of the aedeagus, and the divided female tergite IX, *Lathrobium kuan* belongs to the *Lathrobium fissispinosum* group, which was previously known from Shaanxi, Gansu, Hubei, Guizhou and Sichuan. The new species is distinguished from the other representatives of this group by the chaetotaxy and shapes of the male sternites VII and VIII, the strongly asymmetrical ventral process and the presence of two (rather than one) sclerotized spines in the internal sac of the aedeagus. For illustrations of the species of the *Lathrobium fissispinosum* group see Assing (2013b, in press b) and Peng et al. (2013b, 2014b).

#### Etymology.

The specific name is the Chinese adjective “kuan” (broad). It refers to the broad ventral process of the aedeagus of this species, when compared with the other species known from Dayao Mountains.

#### Distribution and natural history.

The type locality is situated in the Shengtang Shan to the southwest of Jinxiu, central Guangxi. The specimens were sifted from leaf litter in a beech forest at an altitude of 1,160 m.

### Lathrobium leii

Taxon classificationAnimaliaColeopteraStaphylinidae

Peng & Li sp. n.

http://zoobank.org/135F9705-E94F-4E4D-B93D-0F663999A135

Figs 4C , 7

#### Type material.

Holotype: ♂, labelled ‘China: Guangxi Prov., Jinxiu Hsien, Shengtang Shan, 23°57'37"N, 110°06'46"E, 1300 m, 21.VII.2014, Peng, Song, Yan & Yu leg.’ (SNUC). Paratypes: 1 ♀, same label data as holotype (SNUC).

#### Description.

Measurements (in mm) and ratios: BL 6.61–6.67, FL 3.11–3.34, HL 0.83–0.87, HW 0.82–0.88, AnL 1.66–1.68, PL 1.12–1.14, PW 0.85–0.90, EL 0.65–0.68, AL 1.26, HL/HW 0.99–1.01, HW/PW 0.96–0.98, HL/PL 0.74–0.76, PL/PW 1.27–1.32, EL/PL 0.58–0.60.

Habitus as in Fig. 4C. Body light brown, legs yellowish brown, antennae light brown.

Head approximately as long as broad; punctation coarse and moderately sparse, distinctly sparser in median dorsal portion; interstices with shallow microreticulation; eyes small and composed of approximately 35 ommatidia.

Pronotum with weakly convex lateral margins in dorsal view; punctation similar to that of head; impunctate midline broad; interstices without microsculpture.

Elytral punctation moderately dense and defined. Hind wings completely reduced. Protarsi without pronounced sexual dimorphism.

**Figure 7.**
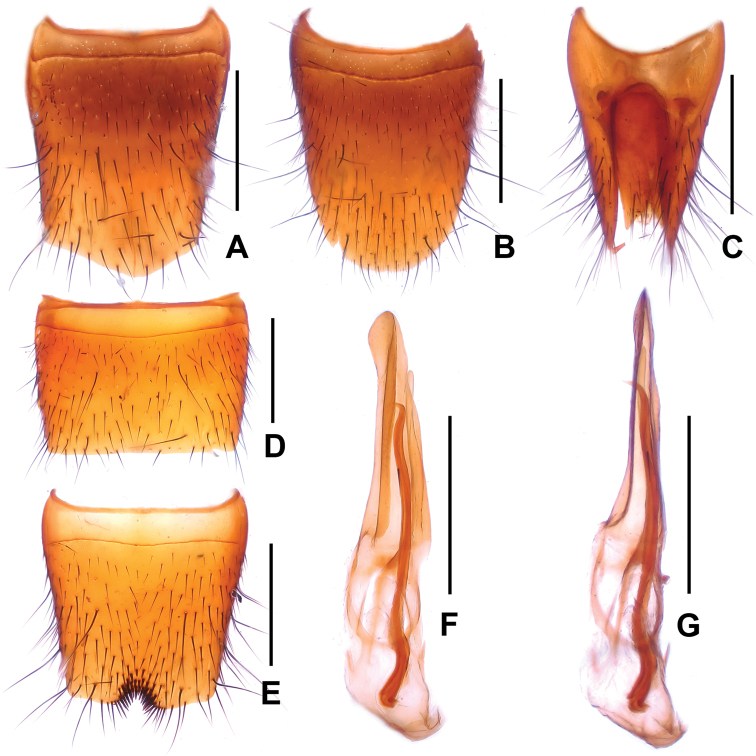
*Lathrobium leii*. **A** female tergite VIII **B** female sternite VIII **C** female tergites IX–X **D** male sternite VII **E** male sternite VIII **F** aedeagus in ventral view **G** aedeagus in lateral view. Scale bars: 0.5 mm.

Abdomen with fine and moderately dense punctation, that of tergite VII somewhat sparser than that of anterior tergites; interstices with shallow microsculpture; posterior margin of tergite VII without palisade fringe; tergite VIII without sexual dimorphism, posterior margin (Fig. 7A) asymmetricalally obtusely angled.

Male. Sternites III-VI unmodified; sternite VII (Fig. 7D) with truncate posterior margin and without distinctly modified setae; sternite VIII (Fig. 7E) with very shallow postero-median impression, this impression with short dark setae posteriorly, posterior excision small and weakly asymmetrical; aedeagus as in Figs 7F, G; ventral process long and slender; dorsal plate with very long moderately sclerotized apical portion and short weakly sclerotized basal portion; internal sac with a long sclerotized spine.

Female. Posterior margin of sternite VIII (Fig. 7B) convex and with moderately dense micropubescence; tergite IX (Fig. 7C) without median suture; tergite X (Fig. 7C) 4.7 times as long as antero-median portion of tergite IX.

#### Comparative notes.

Based on the different general morphology of the aedeagus, *Lathrobium leii* represents a different lineage than the other species recorded from Dayao Mountains. It is additionally distinguished from them by smaller body size, yellowish brown legs, the chaetotaxy of the male sternite VIII, and a slender aedeagus with a long sclerotized spine.

#### Etymology.

The species is dedicated to Yu-Yang Lei, who supported us on our field trips.

#### Distribution and natural history.

The type locality is situated in the Shengtang Shan to the southwest of Jinxiu, central Guangxi. The specimens were sifted from leaf litter and humus in a rhododendron forest at an altitude of 1,300 m, together with *Lathrobium shengtangshanense*.

### Lathrobium dayaoshanense

Taxon classificationAnimaliaColeopteraStaphylinidae

Peng & Li, 2012

#### Material studied.

China: Guangxi: 2 ♂♂, 3 ♀♀, Jinxiu Hsien, Houzi Shan, Yinshan Station, 24°10'01"N, 110°14'38"E, 1200 m, 10.VII.2014, Peng, Song, Yan & Yu leg. (SNUC); 1 ♀, Jinxiu Hsien, Changtanghe, 24°16'00"N, 110°13'29"E, 860 m, 15.VII.2014, Peng, Song, Yan & Yu leg. (SNUC); 1 ♀, Jinxiu Hsien, Qigongli, 24°09'07"N, 110°12'29"E, 1300 m, 16.VII.2014, Peng, Song, Yan & Yu leg. (SNUC); 1 ♂, Jinxiu Hsien, Laoshan, 24°07'02"N, 110°11'51"E, 950 m, 26.VII.2014, Peng, Song, Yan & Yu leg. (SNUC).

#### Comment.

The above material was collected in several localities in the region to the north, east, and northwest of Jinxiu, central Guangxi. The specimens were sifted from deep leaf litter layers in mixed forests at altitudes of 860–1,300 m.

### Lathrobium shengtangshanense

Taxon classificationAnimaliaColeopteraStaphylinidae

Peng & Li, 2012

#### Material studied.

China: Guangxi: 5 ♂♂, 4 ♀♀, Jinxiu Hsien, Shengtang Shan, 23°57'37"N, 110°06'46"E, 1300 m, 21.VII.2014, Peng, Song, Yan & Yu leg. (SNUC).

#### Comment.

*Lathrobium shengtangshanense* has been recorded only from the Shengtang Shan in Guangxi.

### Lathrobium sp.

Taxon classificationAnimaliaColeopteraStaphylinidae

#### Material studied.

China: Guangxi: 1 ♀, Jinxiu Hsien, Shiliugongli, 24°08'25"N, 110°15'38"E, 960 m, 13.VII.2014, Peng, Song, Yan & Yu leg. (SNUC).

#### Comment.

The above micropterous female represents an undescribed species distinguished from the other species known from Dayao Mountains by somewhat smaller body size, the slender pronotum and the female secondary sexual characters.

## Supplementary Material

XML Treatment for Domene (Macromene) hei

XML Treatment for Domene (Macromene) chenae

XML Treatment for Lathrobium jinxiuense

XML Treatment for Lathrobium kuan

XML Treatment for Lathrobium leii

XML Treatment for Lathrobium dayaoshanense

XML Treatment for Lathrobium shengtangshanense

XML Treatment for Lathrobium sp.
